# Flow Synthesis of Pharmaceutical Intermediate Catalyzed by Immobilized DERA: Comparison of Different Immobilization Techniques and Reactor Designs

**DOI:** 10.3390/molecules30112276

**Published:** 2025-05-22

**Authors:** Dino Skendrović, Anita Šalić, Ivan Karlo Cingesar, Marta Pinčić, Ana Vrsalović Presečki

**Affiliations:** 1 Faculty of Chemical Engineering and Technology, University of Zagreb, 10000 Zagreb, Croatia; dskendrov@fkit.unizg.hr (D.S.); asalic@fkit.unizg.hr (A.Š.); mpincic@fkit.hr (M.P.); 2 Faculty of Food Technology and Biotechnology, University of Zagreb, 10000 Zagreb, Croatia; ikcingesar@pbf.hr

**Keywords:** immobilization, statins, aldolase, flow millireactors

## Abstract

The enzymatic synthesis of statin intermediates offers a sustainable alternative to traditional multistep chemical methods. This study investigates the continuous flow synthesis of statin precursors in a millireactor using 2-deoxy-D-ribose-5-phosphate aldolase (DERA) immobilized on mesoporous silica foam (MCF) and magnetic nanoparticles (MNPs). Two types of flow millireactors, a fixed bed millireactor for MCF and a fluidized bed millireactor for MNP, were designed. Key performance indicators including conversion, selectivity, yield, and productivity were analyzed and compared with the batch reactor results. The MNP-based fluidized bed millisystem demonstrated superior conversion (97.78%) and yield (95.85%) under optimized conditions, outperforming both batch and MCF-based millisystems. This work highlights the importance of optimizing immobilization techniques and reactor configurations to enhance enzyme stability and catalytic efficiency in continuous biocatalytic processes, particularly for pharmaceutical applications.

## 1. Introduction

Biocatalysis, as a method for accelerating chemical reactions, is increasingly being used in various industrial processes due to its many advantages. This mainly involves carrying out highly stereoselective reactions under mild temperature, pressure and pH conditions, which are also aligned with the principles of green chemistry [1,2]. In the pharmaceutical industry, the synthesis of statins in particular is of great importance for the pharmacological treatment of high LDL cholesterol levels and associated cardiovascular diseases [3,4,5]. Conventional methods of synthesizing statins involve chemical reactions that are carried out in a lengthy, multi-step process under extreme pressure and temperature conditions. The synthesis is further complicated by the presence of two chiral centers in the molecular structure of statins. Due to these challenges, there is a need to develop environmentally friendly and economically sustainable methods for statin production [5,6,7]. Of these methods, one of the most promising at present is the use of 2-deoxy-D-ribose-5-phosphate aldolase (DERA, EC 4.1.2.4), which catalyzes a sequential aldol addition between chloroacetaldehyde (CAA) and acetaldehyde (AA), leading to the synthesis of a lactol intermediate with two chiral centers, which is then used in further steps to synthesize the drugs atorvastatin and rosuvastatin [8,9,10]. Although the DERA enzyme has considerable advantages, several key challenges stand in the way of its large-scale application. The main obstacles include high production costs and sensitivity to high concentrations of reaction substrates and intermediates, which can lead to inhibition and deactivation [11,12]. However, various strategies can help to mitigate these problems, either by modifying the enzyme itself through techniques such as directed evolution and site-directed mutagenesis, or by increasing its stability during the reaction through various immobilization methods [12,13,14,15]. Among these approaches, immobilization, specifically covalent immobilization, can offer significant advantages for the use of enzymes in industry and especially for continuous reactions, as it improves their stability and allows easier handling and reusability due to the heterogeneous nature of the immobilized enzyme [16,17,18,19].

When selecting carriers for covalent immobilization, it is important to consider the cost and the thermal and mechanical stability of the material as well as the need for a large specific surface area that can be easily chemically modified with various activating agents to which the enzyme is subsequently bound [16,17,18]. These requirements are met by materials with an inorganic structure, such as various forms of silica and metal oxides. Silica, especially in its mesoporous form (pores in the 2–50 nm range), such as mesocellular silica foam (MCF) is an ideal candidate and is widely used in industry. It has also been used with some success for the immobilization of DERA [20,21]. This type of silica has a large specific surface area and pore volume, the ability to easily define the pore size and structure during synthesis, and its surface can be easily modified due to the high concentration of silanol groups [22,23,24,25]. For metal oxides, such as magnetite (Fe_3_O_4_), the most important property is its paramagnetism, which greatly facilitates the manipulation of the particles by an external magnetic field and is of great advantage in the separation and isolation of particles from the reaction mixture. As magnetite tends to oxidize and agglomerate, it must be coated with a protective layer, usually silicon dioxide, which also provides a suitable surface for the binding of various activation groups [26,27,28].

In this study, the synthesis of the statin precursor was carried out in two different types of millireactor systems. While batch reactors are generally the most common type of reactor used in enzymatic synthesis processes, there is a steadily growing interest in the application of continuous flow reactors [29,30], especially millireactors. The main advantages of using continuous millireactor systems are efficiency and scalability, numbering-up, high surface-to-volume ratio, as well as the possibility of the finer control of reaction conditions (temperature, residence time, concentration), less waste generation and less mechanical stress on the enzyme due to the lack of mixing, resulting in a more efficient and stable alternative to enzymatic batch synthesis [29,30,31,32]. The application of a continuous system for the synthesis of statin precursors has been sparse, with one paper by Grabner et al. [19], in which the authors focused on the reaction in a fixed bed reactor with DERA immobilized on an alginate Loofa matrix, and another by Hindges née Bramski et al. [33] in which the authors used a packed bed reactor and focused on the preparation and isolation of the intermediate in the double aldol addition leading to the statin precursors.

**Figure 1:**
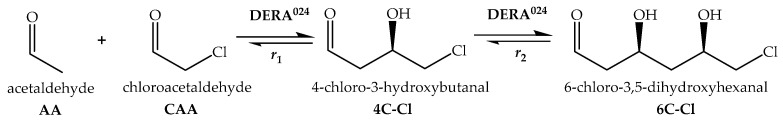
Double aldol addition reaction for the synthesis of statin precursors.

The reaction of interest for this study is the double aldol addition of acetaldehyde and chloroacetaldehyde catalyzed by DERA and which is shown in Figure 1. In our previous work we optimize covalent immobilization DERA on mesoporous silica foam and magnetic nanoparticles (MNPs) [34,35]. Both immobilized enzymes show superior stability in multicycle experiments over free enzymes. This motivated us to explore the possibility of its application in a flow setup. Based on their support characteristics, two different continuous flow reactors were chosen: A 3D-printed packed bed millireactor with MCF as support and a fluidized bed millireactor in an oscillating magnetic field for DERA immobilize on MNP. The study provides a detailed process engineering perspective, examining key process indicators (conversion, selectivity, yield, productivity) under varying operational conditions. Moreover, it demonstrates, for the first time, the successful implementation of a fluidized bed millireactor with magnetically retained DERA catalysts under continuous flow for prolonged operational periods with high performance. This comparative approach not only identifies the most effective configuration but also delivers new insights into the hydrodynamic and catalytic behavior of immobilized DERA in continuous systems, offering valuable guidance for future scale-up and industrial application.

## 2. Results and Discussion

The DERA enzyme offers significant advantages, primarily due to its ability to catalyze the formation of two stereocenters in a single step from relatively simple aldehyde substrates [36]. However, the major challenge remains the limited stability of the enzyme. In previous studies, we addressed this issue by optimizing the covalent immobilization of DERA^024^ on both magnetic carriers and mesoporous silica. The use of different activating agents not only enhanced the enzyme’s stability but also led to hyperactivation—a rare phenomenon attributed to the favorable conformational changes induced during covalent binding [34,35].

Immobilization of the enzyme not only contributes to increased stability but also facilitates its application in flow processes. In this study, we investigated the performance of these optimized immobilized enzymes in continuous flow millisystems. We selected millireactor systems with volumes under 1 mL, because such systems offer numerous advantages, including improved mass and heat transfer, better control of process parameters (such as temperature and flow rate), and straightforward production increase, by increasing the number of reactors (numbering up) instead of increasing the reactor volume [37].

Two types of continuous millireactor systems for heterogeneous catalysis were employed. Reactions using DERA^024^ immobilized on MCF were carried out in three fixed bed millireactors, while MNPs were applied in a fluidized bed millireactor, with catalyst retention achieved via an external magnetic field. In all experiments, we investigated the impact of two key parameters, substrate concentration and flow rate, which determine residence time and significantly influence process efficiency [38].

### 2.1. Statin Precursor Synthesis in a Fixed Bed Millireactor

**Figure 2:**
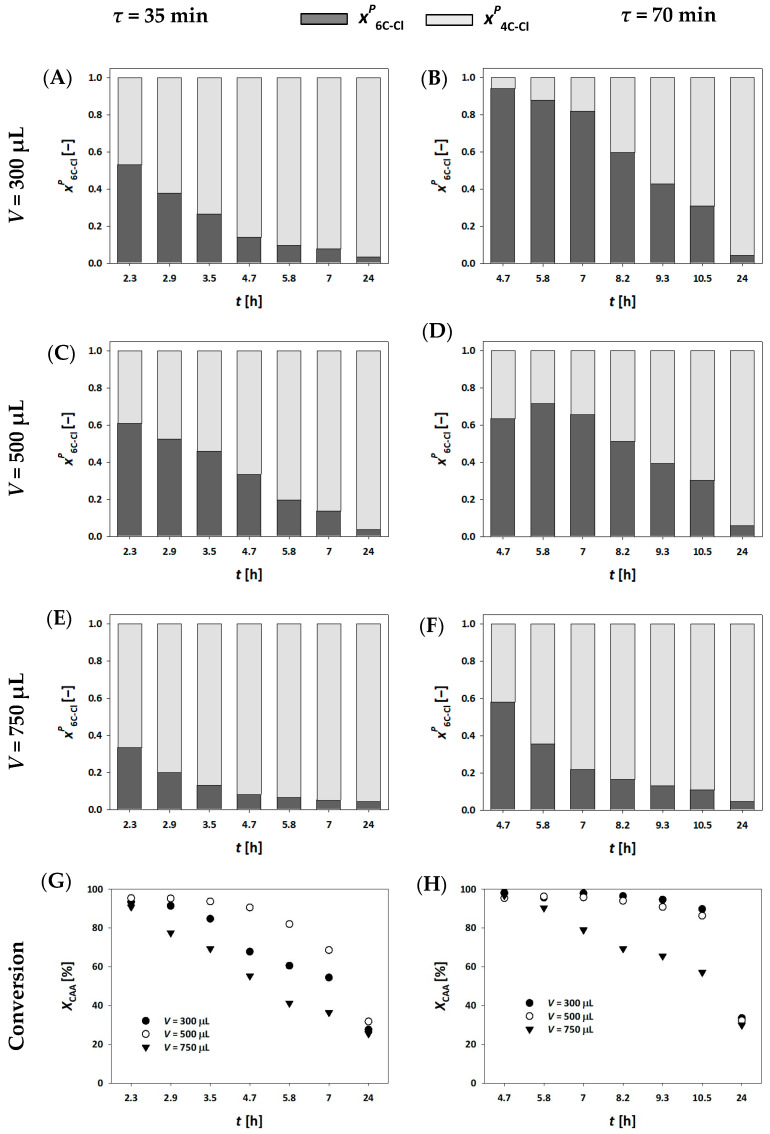
Molar share of single (4C-Cl) and double (6C-Cl) aldol adducts (**A**–**F**), and chloroacetaldehyde (CAA) conversion (**G**,**H**) during the DERA^024^-catalyzed double aldol addition in a fixed bed millireactor, with the enzyme immobilized on MCF (*c*_AA_ = 100 mmol L^−1^, c_CAA_ = 50 mmol L^−1^, phosphate buffer 0.1 mol L^−1^, pH 6, 25 °C, *γ*_DERA024_ = 4.10 mg mL^−1^).

The DERA^024^ enzyme immobilized on mesoporous silica [34] was applied in fixed bed millireactors, a setup that has been demonstrated for DERA enzyme in previous studies [19,33,38]. Three different millireactor volumes were used to investigate the effect of characteristic millireactor dimensions (length, width and depth) on key process indicators. Flow rates were adjusted so that residence times of 35 and 70 min were the same for all tested millireactors, while initial substrate concentrations remained the same. Samples were collected at the reactor outlet during each experiment and analyzed using the Bradford assay to monitor potential enzyme leaching. No detectable enzyme loss was observed throughout the experiments. Figure 2A–F present the molar fractions of the single and double aldol adducts, while Figure 2G,H display conversion data for all three millireactor volumes at different residence times.

Despite the differences in reactor volumes, steady-state conditions were not achieved in terms of product concentrations. However, conversion values remained stable at a 70 min residence time (Figure 2H) for the smaller millireactor volumes (300 μL and 500 μL) during the initial 8 h of operation. Interestingly, under these conditions, the concentration of the double aldol adduct (6C-Cl) decreased with time. At the same time, the concentration of the intermediate (4C-Cl) continued to increase even after the conversion to the final product (6C-Cl) had stopped. This behavior suggests that enzyme inactivation over time may have hindered the progression of the second aldol reaction under constant flow conditions.

Despite the challenges outlined above, the experimental results demonstrate that the described setup is still suitable for the production of single aldol adducts. These molecules, containing a single chiral center, are of particular interest to the pharmaceutical industry, where they serve as valuable intermediates for the synthesis of targeted active pharmaceutical ingredients [33].

An interesting observation emerged when comparing the effects of millireactor volume and flow rate on enzyme performance. At higher flow rates, corresponding to shorter residence times, the decline in conversion was slowest in the medium-sized millireactor and fastest in the largest millireactor (Figure 2G). Similarly, the largest millireactor again showed the poorest performance under conditions of extended residence time (Figure 2H). In order to achieve equivalent residence times in the millireactor with the larger volume, significantly higher flow rates were required compared with the smaller reactors. These higher flow rates likely generated increased shear forces, which contributed to accelerated enzyme deactivation [39]. These findings highlight the complex interplay between millireactor hydrodynamics and biocatalyst stability. In particular, they emphasize the importance of optimizing the millireactor design and operational parameters, to preserve enzyme activity and ensure consistent product formation [33].

Several strategies can be used to mitigate enzyme inactivation in the DERA flow system, including the extension of residence time, reduction of initial substrate concentrations, or an increase in enzyme concentration. It is well documented that DERA is particularly sensitive to the concentration of the intermediate product, which, in contrast to the final product, contributes significantly to enzyme inactivation [36]. As reported by Švarc et al. [36], low enzyme concentrations in the reactor correlate with a higher rate of deactivation. This behavior can be attributed to the kinetics of the two-step aldol reaction in which the first reaction proceeds faster than the second, which leads to the accumulation of the reactive intermediate if the enzyme concentration is insufficient. This intermediate accumulation accelerates enzyme inactivation.

**Figure 3:**
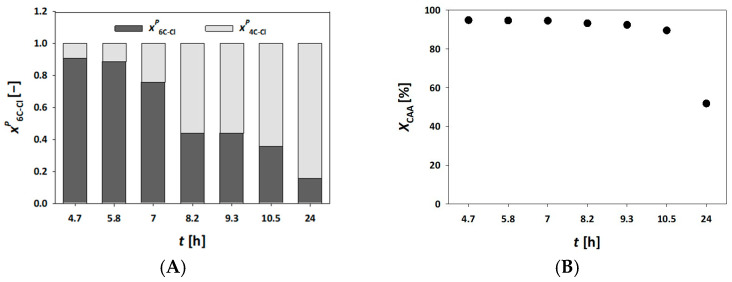
Molar share of single (4C-Cl) and double (6C-Cl) aldol adducts (**A**), and chloroacetaldehyde conversion (**B**) during the DERA^024^-catalyzed double aldol addition in a fixed bed reactor, with the enzyme immobilized on MCF (*V* = 500 µL, *τ* = 70 min, *c*_AA_ = 50 mmol L^−1^, *c*_CAA_ = 25 mmol L^−1^, phosphate buffer 0.1 mol L^−1^, pH 6, 25 °C, *γ*_DERA024_ = 4.10 mg mL^−1^).

In our system, all millireactors were already fully packed with carrier material, making it impractical to further increase the enzyme loading. Additionally, a residence time of 70 min was the upper practical limit for maintaining productivity and continuously monitoring the reaction throughout the working day. Given these limitations, we also explored the influence of substrate concentration on the process outcome. The most feasible adjustment was to reduce the substrate concentrations in the feed to *c*_AA_= 50 mmol L⁻^1^ and *c*_CAA_= 25 mmol L⁻^1^. As shown in Figure 3, lower substrate concentrations significantly improved enzyme stability. Under these conditions, the concentration of the final product, 6C-Cl, reached a steady state within the first two hours of the reaction, achieving a maximum concentration of c_6C-Cl_ = 22.90 mmol L⁻^1^ and a yield of *Y* = 91.61%. Notably, the conversion remained stable for up to 6 h.

These findings reinforce the hypothesis that minimizing intermediate accumulation in the system leads to enhanced enzyme stability, thereby extending the operational lifetime and efficiency of the biocatalytic process [36].

### 2.2. Statin Precursor Synthesis in a Fluidized Bed Millireactor

In a previous study, we optimized the immobilization of the DERA^024^ enzyme on MNP [35]. This immobilization strategy resulted in a higher degree of hyperactivation compared with MCF supports, 179% versus 139%, respectively [34,35]. Furthermore, the DERA immobilized on MNP demonstrated superior stability in reuse experiments.

Due to the nanoscale size and unique behavior of the MNPs, we selected a fluidized bed millireactor configuration for their application in flow processes. This choice helped to circumvent several common challenges associated with the use of such fine catalysts in fixed bed millireactors, including clogging or channel, poor flow distribution, increased pressure drop, inadequate mass transfer and catalytic activity loss due to particle aggregation [40]. The fluidized bed millireactor, coupled with an external magnetic field, effectively retains the catalyst within the reactor and prevents leaching [41]. However, a notable limitation of this system is that it cannot be filled with as much catalyst as packed bed configurations, which may constrain the overall productivity of the system [42].

**Figure 4:**
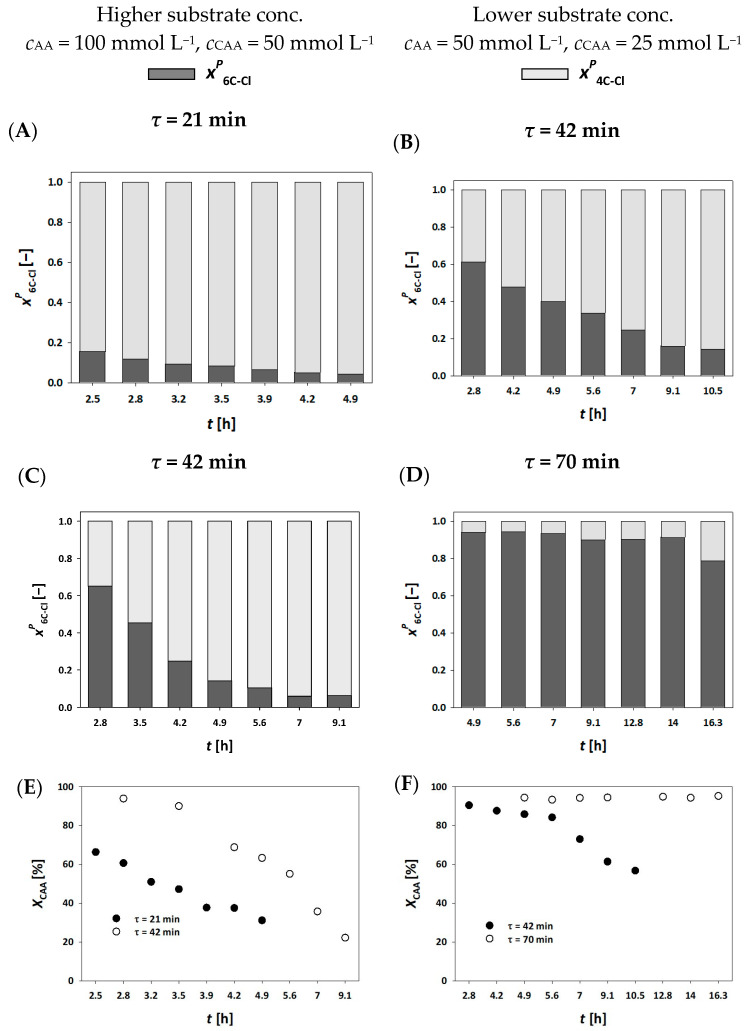
Molar share of single (4C-Cl) and double (6C-Cl) aldol adducts (**A**–**D**), and chloroacetaldehyde (CAA) conversion (**E**,**F**) during the DERA^024^-catalyzed double aldol addition in a fluidized bed reactor, with the enzyme immobilized on MNP (*V* = 310 μL, phosphate buffer 0.1 mol L^−1^, pH 6, 25 °C, *γ*_DERA024_ = 4.52 mg mL^−1^).

Figure 4A,C,E presents the results of experiments conducted in a fluidized bed millireactor at residence times of 21 and 42 min, using higher substrate concentrations. Based on the obtained process parameters, these residence times proved inadequate, as they resulted in very low conversion and rapid enzyme inactivation (Figure 4E). In both cases, a similar trend to the fixed bed millireactor using mesoporous silica was observed, i.e., the single aldol adduct 4C-Cl remained the dominant product. At a residence time of 42 min, the conversion to 6C-Cl was slightly improved compared with *τ* = 21 min, but the overall conversion rates remained low.

Interestingly, when comparing the experiment at *τ* = 42 min in the fluidized bed millireactor (Figure 4C,E) to the fixed bed experiment with MCF at *τ* = 35 min and a millireactor volume of 300 µL (Figure 2A,G), it appears that the enzyme immobilized on MNP underwent faster deactivation. This observation is in contrast to previous results of reuse studies, where the enzyme immobilized on magnetic nanoparticles exhibited higher stability [34,35]. This discrepancy suggests that the millireactor configuration and hydrodynamic conditions may significantly influence enzyme performance, even when immobilization stability is optimized [33].

To improve the results, further experiments were carried out using longer residence times and reduced substrate concentrations since initial experiments indicated that both residence times and substrate concentrations were suboptimal as they led to insufficient conversion. Therefore, half the original substrate concentration and longer residence times were used in experiments, as shown in Figure 4B,D,F. As evident from the results at *τ* = 42 min (Figure 4D), a steady state was achieved for 4 h, resulting in consistent conversion to the single aldol adduct (Figure 4F). However, enzyme inactivation still occurred, halting further conversion to the final product.

To address this, the residence time was increased to 70 min while maintaining the reduced substrate concentration. Under these conditions, as presented in Figure 4D,F, the reaction reached a near-ideal performance, with a sustained steady state of 15 h and a final 6C-Cl yield of 95%. Thus, the enzyme immobilized on MNP outperformed that immobilized on MCF (Figure 3), which aligns with previous results obtained from reusability tests [34,35]. As previously noted, this performance advantage was not observed under conditions of higher flow rates or shorter residence times. The enzyme DERA is highly sensitive to aldehydes i.e., substrates, and is able to maintain steady-state conditions and prolong biocatalyst life. Thus, it is necessary to optimize the concentrations of AA and CAA in the feed stream, as demonstrated in the experimental results (Figure 3B and Figure 4F).

Thus, it can be concluded that successful implementation of immobilized enzymes in both fixed bed and fluidized bed millireactors requires careful experimental optimization. This necessity arises from the complex interplay of factors such as mass and energy transfer, shear forces, and magnetic field effects on the catalyst [41]. Therefore, another significant advantage of millireactor systems is their capacity increase through the numbering-up principle, which eliminates the need for the time-consuming re-optimization that is usually required when scaling-up reactor volumes [43].

### 2.3. Comparison of the Results of Batch and Continuous Millireactor Types for the Production of Statin Precursors

Table 1 summarizes the calculated process indicators for the different reactor types and catalyst carriers examined in this study. The batch reactor results, taken from our previous experiments [34,35], serve as a reference for comparison. While the productivity of the DERA^024^ enzyme immobilized on MCF and MNP in batch reactors was similar, the MNP-based system showed clear advantages. Specifically, in the batch reactor the use of MNP-immobilized DERA^024^ led to significantly higher selectivity, conversion, and overall product yield over MCF-DERA^024^.

To intensify the biocatalytic process, additional experiments were performed using flow millireactors configured with both fixed bed and fluidized bed systems containing the immobilized enzyme. Two types of flow reactors were used because of the distinct particle characteristic of carrier used for DERA024 immobilization. MCF particles are relatively large and porous, resulting in a high-volume occupation, which can limit the achievable biocatalyst concentration in a fluidized bed reactor. Conversely, the application of MNPs in a packed bed reactor may lead to system clogging and significant pressure drops due to their small particle size. Nonetheless, the use of immobilized enzymes in both reactor systems is well established [44].

Among flow setups, the best-performing configuration employed the DERA^024^ enzyme immobilized on MNPs within a fluidized bed millireactor. This system achieved superior process metrics, including a high selectivity (*S* = 16.34), along with excellent conversion (*X* = 97.78%) and yield (*Y* = 95.85%). These results surpass those achieved with MCF-based catalysts and batch systems. To enable a direct comparison of the performance of immobilized and free DERA^024^ under identical process conditions, simulations were conducted for the free enzyme in a continuous membrane reactor using the kinetic and stability parameters reported by Švarc et al. [45] (Figures S1 and S2). The results show that steady-state conditions could not be achieved with the free enzyme and that CAA conversion is significantly lower, highlighting the significant improvement in stability and activity provided by enzyme immobilization.

In summary, while the batch reactor demonstrated higher productivity, the flow millireactor system, particularly when combined with MNP-immobilized DERA^024^, offered significantly improved selectivity and yield. Therefore, the choice of reactor type should be guided by the desired process priorities: if productivity is the primary goal, batch reactors are preferable; however, for applications where selectivity and efficiency are critical, flow millireactors with magnetic nanoparticle-based catalysts represent a more advantageous solution.

## 3. Materials and Methods

### 3.1. Chemicals

All materials were used without further purification and were of analytical grade. Acetaldehyde, pluronic P123, 3-aminopropyltriethoxysilane (APTES) and (3-Methylaminopropyl) trimethoxysilane (APTMS) were obtained from Acros Organics (Geel, Belgium). Potassium dihydrogen phosphate, dipotassium hydrogen phosphate, hydrochloric acid (HCl), sodium acetate anhydrous, ethylene glycol and tetraethyl ortosilicate (TEOS) were obtained from Lach-Ner (Neratovice, Czech Republic). o-benzylhydroxylamine hydrochloride was obtained from TCI (Oxford, UK). Bovine serum albumin (BSA), succinic anhydride, Iron (III) chloride hexahydrate (FeCl_3_∙6H_2_O) and chloroacetaldehyde solution (50% (*w*/*w*)) were obtained from Sigma-Aldrich (Schnelldorf, Germany). Ethanol absolute was from Scharlau (Barcelona, Spain). DERA^024^ from *Thermotoga maritima* was obtained from Prozomix (Haltwhistle, UK). Acetonitrile, trifluoracetic acid (TFA) and 1,2,4-Trimethylbenzene were obtained from Fisher Scientific (Loughborough, UK).

### 3.2. Synthesis, Functionalization and Activation of Immobilization Carriers

The synthesis of MCF and MNP was performed as described earlier [34].

Functionalization of MCF was undertaken with APTMS, and MNP with APTES. Activation of MCF was achieved with 10% *v*/*v* succinic anhydride, and MNP with 15% *v*/*v* succinic anhydride. An amount of 1 g Fe_3_O_4_@SiO_2_/MCF was dispersed in 50 mL ethanol, followed by the addition of 3 mL APTMS or APTES. The mixture was stirred at 160 rpm and 30 °C for 24 h under an inert argon atmosphere. The resulting Fe_3_O_4_@SiO_2_/MCF-NH_2_ precipitate was washed with ethanol and dried overnight at 60 °C. For succinic anhydride activation, the Fe_3_O_4_@SiO_2_/MCF-NH_2_ particles were stirred for 2 h at 900 rpm in 1 mL of 0.1 mol L^−1^ potassium phosphate buffer (pH 6) containing 10/15% succinic anhydride under an inert argon atmosphere.

### 3.3. Enzyme Immobilization

Following activation, enzyme immobilization was carried out by mixing different amounts of activated MNP/MCF with 1 mL of a 0.6 mg mL^−1^ DERA (1.92 U/mg; 1 U correspond to the conversion of 1 µmol of CAA per minute) solution in 0.1 mol L^−1^ potassium phosphate buffer (pH 6). The mixture was stirred at 900 rpm at 25 °C for 2 h. After this time, a sample of the supernatant was taken to determine the amount of unbound enzyme using the standard Bradford assay. Based on these measurements, the immobilization yield was calculated using the following formula:

(1) $$Immobilization\;yield\;(\%)=\frac{c_{e}-c_{s}}{c_{e}}$$

where *c_e_* is the initial enzyme concentration (mg mL^−1^) and *c_s_* is the enzyme concentration in the supernatant (mg mL^−1^).

### 3.4. Statin Precursor Synthesis in Packed Bed Millireactor

**Figure 5:**
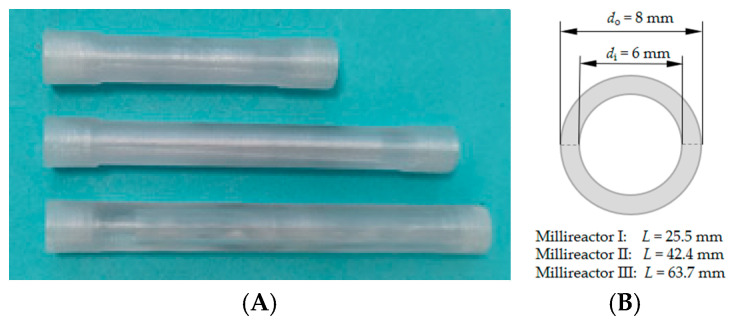
(**A**) Three-dimensionally printed tubular millireactors with volumes of 300, 500 and 750 μL and (**B**) characteristics of the millireactors.

MCF was tested as a carrier for immobilization in a fixed bed millireactor to evaluate the effects of different flow rates on the formation of the intermediate 4C-Cl and the final product 6C-Cl. Tubular millireactors (Figure 5) with volumes of 300 μL (*d*_o_ = 8 mm, *d*_i_ = 6 mm, *l* = 25.5 mm), 500 μL (*d*_o_ = 8 mm, *d*_i_ = 6 mm, *l* = 42.4 mm) and 750 μL (*d*_o_ = 8 mm, *d*_i_ = 6 mm, *l* = 63.7 mm) were designed in Autodesk Fusion computer-aided design (CAD) software. Relevant dimensions are listed in brackets for all three reactors. The reactors were then manufactured using Original Prusa i3 MK3s+ (Prusa Research, Prague, Czech Republic) which uses fused filament fabrication (FFF) technology. Polyethylene terephthalate glycol (PETG) filament with a diameter of 1.75 mm ± 5% from Devil Design (Devil Design Ryszka Mateja Sp. J., Mikołów, Poland) was used to manufacture reactors. In the PrusaSlicer slicing software, the defined settings for 3D printing were 235 °C nozzle temperature and 80 °C heatbed temperature, layer thickness was set to 0.05 mm, and infill was set to 100%.

**Figure 6:**
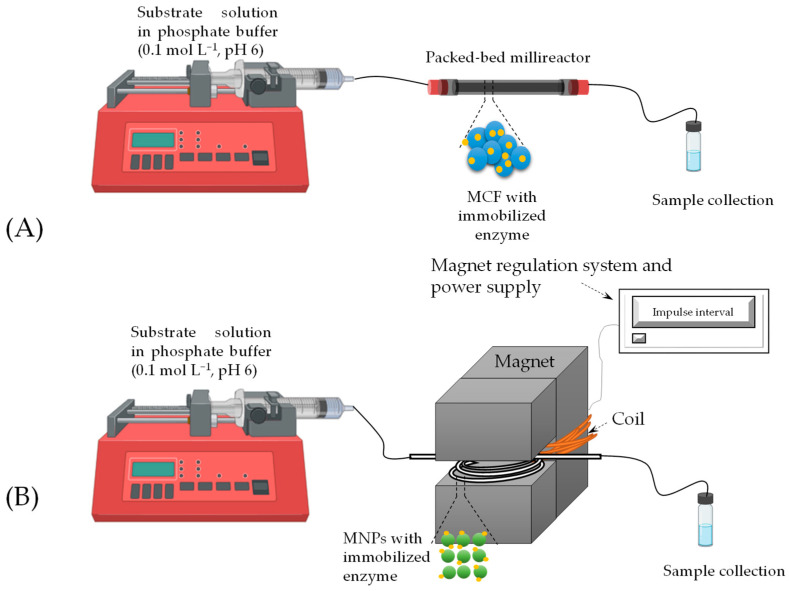
Experimental setup used for statin precursor synthesis in (**A**) packed bed millireactor and (**B**) fluidized bed millireactor inside an electromagnetic field.

A mixture of MCF with immobilized enzyme (*immobilization yield* = 68.33%) was first introduced into the reactors using a piston pump. The reactor inlet was then connected to another piston pump (Figure 6A), which pumped a substrate solution with a specific concentration in phosphate buffer (0.1 mol L^−1^, pH 6) at different flow rates (Table 2). Samples were taken after 4 residence times, 4*τ*, have passed, assuming steady-state conditions had been reached. The enzyme concentration in each millireactor was 4.10 mg mL^−1^. The working volume (*V_R_*, Table 2) was determined by the difference in weight of the reactor with the MCF-enzyme carrier with a reaction medium and the reactor with MCF-enzyme. The conditions for all reactions are listed in Table 2. Based on the measured values, key process indicators were calculated, including selectivity (*S*, Equation (2)), conversion (*X*, Equation (3)), productivity (*Pr*, Equation (4)) and yield (*Y*, Equation (5)).

(2) $$S=\frac{c_{6C-Cl}}{c_{4C-Cl}}$$

(3) $$X=\frac{c_{CAA,0}-c_{CAA}}{c_{CAA,0}}$$

(4) $$Pr=\frac{c_{6C-Cl}}{\tau }$$

(5) $$Y=\frac{c_{6C-Cl}}{c_{CAA,\;0}}$$

### 3.5. Statin Precursor Synthesis in a Fluidized Bed Millireactor

MNPs were tested as carriers for immobilization in a fluidized bed tubular reactor to investigate the effects of different flow rates on the formation of the intermediate 4C-Cl and the final product 6C-Cl. A Teflon tube (*d*_o_ = 5 mm, *l* = 15.8 mm) with a volume of 310 μL was used as the tubular millireactor. A mixture of MNPs with immobilized enzyme (*immobilization yield* = 75.33%) was added to the tube using a syringe, after which the reactor was placed in an electromagnet with a variable magnetic field strength of *H* = 0.5 T and a frequency of 0.5 Hz. A schematic representation of the reaction is shown in Figure 6B. The reactor was then connected to a piston pump, which introduced a substrate solution of a specific concentration into phosphate buffer (pH 6, 0.1 mol L^−1^) at different flow rates (Table 3). As with the fixed bed reactor, samples were taken after four residence times had passed, assuming that steady-state conditions were achieved. The enzyme concentration in the reactor was 4.52 mg mL^−1^. The working volume (*V_R_*, Table 3) was calculated using the same method as described above in a fixed bed millireactor. The conditions for all reactions are listed in Table 2. Based on the obtained values, the process indicators were calculated (Equations (2)–(5)).

### 3.6. Analytical Methods

Substrate and product concentrations were analyzed by HPLC with a Phenomenex Kinetex column as described earlier [34,35]. Protein concentrations were determined spectrophotometrically using Bradford assay [34,35].

## 4. Conclusions

This study demonstrates the viability of continuous flow millireactors using immobilized DERA for the efficient synthesis of statin precursors. The immobilization of DERA on mesoporous silica and magnetic nanoparticles provided enhanced enzyme stability and enabled the operation of fixed bed and fluidized bed reactors, respectively. Among the systems tested, the fluidized bed reactor with MNP-immobilized enzyme yielded the highest selectivity and conversion, although fixed bed reactors exhibited slightly higher productivity. Notably, enzyme inactivation was mitigated through extended residence times and reduced substrate concentrations. The results underscore the critical role of reactor design, flow dynamics, and immobilization strategy in optimizing biocatalytic processes. Flow systems, particularly those utilizing magnetic nanoparticles, offer scalable and efficient alternatives to batch synthesis, especially when selectivity and operational longevity are prioritized.

## Figures and Tables

**Table 1:** Comparison of process indicators for batch and flow reactors.

Reactor Mode	Catalyst	*t* (*τ*) (min)	*γ*_DERA_^024^ (mg mL^−1^)	*S* (-)	*X*_CAA, max_ (%)	*Pr*_6C-Cl_, *c*_AA_ (mmol L^−1^ min^−1^)	*Y* (%)
Batch	MCF-DERA^024^	40	3	3.6	92.25	0.87	76.10
	MNP-DERA^024^	40	3	5.5	98.30	0.88	90.20
Flow—fixed bed	MCF-DERA^024^	70	4.10	9.72	94.52	0.37	91.60
Flow—fluidized bed	MNP-DERA^024^	70	4.52	16.34	97.78	0.33	95.85

**Table 2:** Process conditions during the reaction in the packed bed millireactor.

Millireactor	Total Volume, *V* (μL)	Working Volume, *V_R_* (μL)	Flow, *Q* (μL min^−1^)	Residence Time, *τ* (min)	AA Concentration, *c*_AA_ (mmol L^−1^)	CAA Concentration, *c*_CAA_ (mmol L^−1^)
R1-1	300	175	5	35	100	50
R1-2	300	175	2.5	70	100	50
R2-1	500	290	8.33	35	100	50
R2-2	500	290	4.16	70	100	50
R3-1	750	438	12.5	35	100	50
R3-2	750	438	6.24	70	100	50
R3-3	750	438	4.16	70	50	25

**Table 3:** Process conditions during the reaction in the fluidized bed millireactor.

Millireactor	Total Volume, *V* (μL)	Working Volume, *V_R_* (μL)	Flow, *Q* (μL min^−1^)	Residence Time, *τ* (min)	AA Concentration, *c*_AA_ (mmol L^−1^)	CAA Concentration, *c*_CAA_ (mmol L^−1^)
R1-1	310	106	5	21	100	50
R1-2			2.5	42	100	50
R2-1			5	21	100	50
R2-2			2.5	42	100	50
R3-1			2.5	42	50	25
R3-2			1.5	70	50	25

## Data Availability

Data will be made available on request.

## Supplementary Materials

The following supporting information can be downloaded at: https://www.mdpi.com/article/10.3390/molecules30112276/s1, Scheme S1. Reaction scheme for double aldol addition of acetaldehyde and chloroacetaldehyde driven by free DERA^024^. Figure S1. Comparison of chloroacetaldehyde conversion during the MCF-DERA^024^ and free DERA^024^ catalyzed double aldol addition in a continuous reactor (*V* = 500 µL, *τ* = 70 min, *c*_AA_ = 50 mmol L^−1^, *c*_CAA_ = 25 mmol L^−1^, phosphate buffer 0.1 mol L^−1^, pH 6, 25 °C, *γ*_DERA024_ = 4.10 mg mL^−1^). Figure S2. Comparison of chloroacetaldehyde conversion during the MNP-DERA^024^ and free DERA^024^ catalyzed double aldol addition in a continuous reactor (*V* = 310 µL, *τ* = 70 min, *c*_AA_ = 50 mmol L^−1^, *c*_CAA_ = 25 mmol L^−1^, phosphate buffer 0.1 mol L^−1^, pH 6, 25 °C, *γ*_DERA024_ = 4.52 mg mL^−1^). References [45,46,47] are cited in Supplementary Materials.

## Abbreviations

The following abbreviations are used in this manuscript:

4C-Cl	4-chloro-3-hydroxybutanal
6C-Cl	6-chloro-3,5-dihydroxyhexanal
AA	Acetaldehyde
APTES	3-aminopropyltriethoxysilane
APTMS	(3-methylaminopropyl) trimethoxysilane
CAA	Chloroacetaldehyde
DERA	2-deoxy-D-ribose-5-phosphate aldolase
HCl	Hydrochloric acid
MCF	Mesocellular silica foam
MNP	Magnetic nanoparticles
TEOS	Tetraethyl ortosilicate
