# Supplementary material for: Flow Synthesis of Pharmaceutical Intermediate Catalyzed by Immobilized DERA: Comparison of Different Immobilization Techniques and Reactor Designs
Source: Molecules. 2025 May 22;30(11):2276. doi: 10.3390/molecules30112276 (PMC12156222; doi:10.3390/molecules30112276)
Supplement: Supplementary file 1 [file molecules-30-02276-s001.zip › molecules-3636916-supplementary.pdf]

SUPPLEMENTARY INFORMATION

Flow synthesis of pharmaceutical intermediate catalyzed by immobilized DERA: comparison of different immobilization techniques and reactor designs

Dino Skendrović [1], Anita Šalić [1], Ivan Karlo Cingesar [2], Marta Pinčić [1], Ana Vrsalović Presečki [1*]

1 University of Zagreb, Faculty of Chemical Engineering and Technology, 10000 Zagreb, Croatia; dskendrov@fkit.unizg.hr (D. S.); asalic@fkit.unizg.hr (A.Š.); mpincic@fkit.hr (M.P.); avrsalov@fkit.unizg.hr (A.V.P)

2 University of Zagreb, Faculty of food technology and biotechnology, 10000 Zagreb, Croatia;
ikcingesar@pbf.hr (I.K.C)

* Correspondence: avrsalov@fkit.unizg.hr; Tel.: +385 1 4597 157

1. Comparison of free and immobilized DERA reaction in flow reactors

To compare the behavior of free DERA^{024} in a flow system with immobilized enzymes, the simulations were performed under identical process conditions. The kinetic and deactivation parameters used in the simulation were taken from the study by Švarc et al. [45]. It is important to note that the reaction scheme differs when using the free enzyme (Scheme S1). In addition to the main reaction yielding lactol, a side reaction involving the trimerization of acetaldehyde take place. This side reaction was not detected with either of the immobilized enzyme preparations.

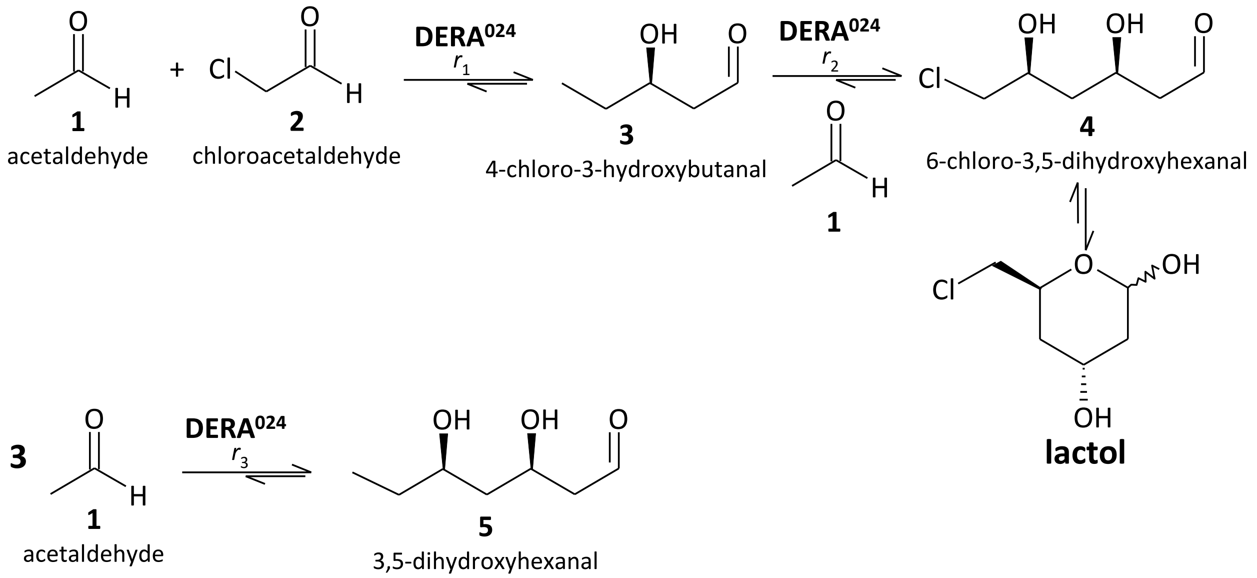

Scheme S1. Reaction scheme for double aldol addition of acetaldehyde and chloroacetaldehyde driven by free DERA^{024}.

Free DERA^{024} is a soluble enzyme, and one viable method for retaining it within the reactor is the use of an ultrafiltration membrane unit, or by integrating the membrane directly into the reactor, as implemented in enzyme ultrafiltration membrane reactors [46]. The reactor model applied for this system assumes no diffusion limitations across the membrane, as described by equations S1–S5 [47].

$$\frac{dc_{\text{acetaldehyde}}}{dt} = \frac{c_{\text{ac,FEED}} - c_{\text{acetaldehyde}}}{\tau} - r_1 - r_2 - 3 \cdot r_3 \tag{S1}$$

$$\frac{dc_{\text{chloroacetaldehyde}}}{dt} = \frac{c_{\text{chlac,FEED}} - c_{\text{chloroacetaldehyde}}}{\tau} - r_1 \tag{S2}$$

$$\frac{dc_{\text{intermediate}}}{dt} = -\frac{c_{\text{intermediate}}}{\tau} + r_1 - r_2 \quad \text{(S3)}$$

$$\frac{dc_{\text{lactol}}}{dt} = -\frac{c_{\text{lactol}}}{\tau} + r_2 \quad \text{(S4)}$$

$$\frac{dc_{\text{side product}}}{dt} = -\frac{c_{\text{side product}}}{\tau} + r_3 \quad \text{(S5)}$$

The comparison of chloroacetaldehyde (CAA) conversion in the flow system using free and immobilized DERA^{024} is presented in Figures S1 and S2.

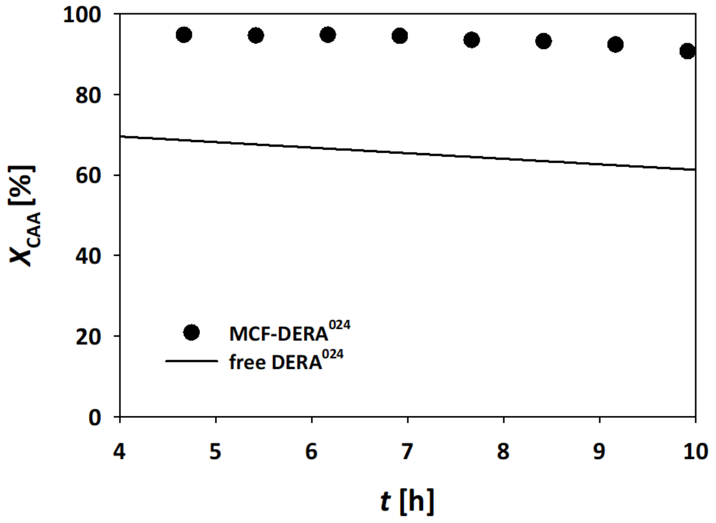

Figure S1. Comparison of chloroacetaldehyde conversion during the MCF-DERA^{024} and free DERA^{024} catalyzed double aldol addition in a continuous reactor (V = 500 μL, τ = 70 min, c_{AA} = 50 mmol L^{-1}, c_{CAA} = 25 mmol L^{-1}, phosphate buffer 0.1 mol L^{-1}, pH 6, 25 °C, γ_{DERA024} = 4.10 mg mL^{-1})

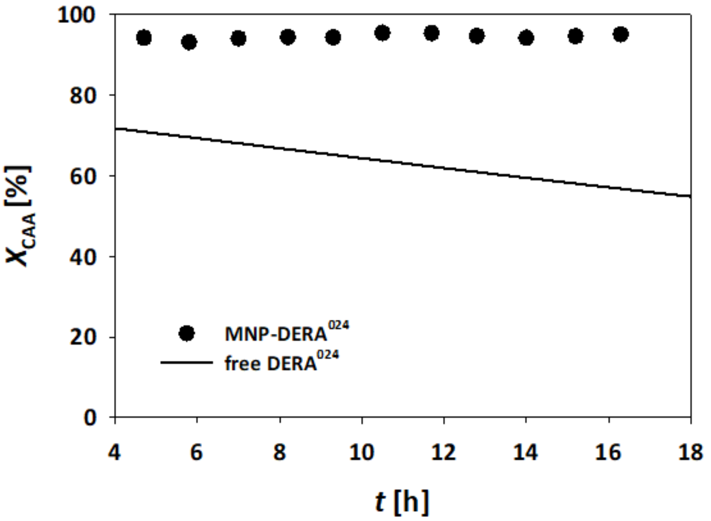

Figure S2. Comparison of chloroacetaldehyde conversion during the MNP-DERA^{024} and free DERA^{024} catalyzed double aldol addition in a continuous reactor (V = 310 µL, τ = 70 min, c_{AA} = 50 mmol L^{-1}, c_{CAA} = 25 mmol L^{-1}, phosphate buffer 0.1 mol L^{-1}, pH 6, 25 °C, $\gamma_{DERA024}$ = 4.52 mg mL^{-1})

References:

[45] Švarc, A., Findrik Blažević, Z., Vasić-Rački, Đ., Charnock, S. J., Vrsalović Presečki, A. A multi-enzyme strategy for the production of a highly valuable lactonized statin side-chain precursor. Chem. Eng. Res. Des. **2020**, 164, 35–45. DOI: 10.1016/j.cherd.2020.09.016

[46] Valinger, D., Vrsalović Presečki, A., Kurtanjek, Ž., Pohl, M., Findrik Blažević, Z., Vasić-Rački, Đ. Continuous enzymatic carboligation of benzaldehyde and acetaldehyde in an enzyme ultrafiltration membrane reactor and laminar flow microreactors. J. Mol. Catal. B Enzym. **2014**, 102, 132–137. DOI: 10.1016/j.molcatb.2014.02.003

[47] Vrsalović Presečki, A., Zelić, B., Vasić-Rački, Đ. Modelling of Continuous L-Malic Acid Production by Porcine Heart Fumarase and Fumarase in Yeast Cells. CABEQ **2009**, 23, 519-525.
